# Salivary biomarkers associated with the progression of disease in people living with HIV: A scoping review

**DOI:** 10.12688/f1000research.50813.1

**Published:** 2021-02-19

**Authors:** Priyanka Prasad, Viola D’Souza, Prasanna Mithra, Raghu Radhakrishnan

**Affiliations:** 1 Manipal College of Dental Sciences, Manipal, Manipal, Karnataka, 576104, India; 2 Prasanna School of Public Health, Manipal Academy of Higher Education, Manipal, Karnataka, 576104, India; 3 Department of Community Medicine, Kasturba Medical College, Mangalore,, Manipal Academy of Higher Education, Karnataka, 575001, India; 4 Department of Oral Pathology, Manipal College of Dental Sciences, Manipal, Manipal Academy of Higher Education, Manipal, Karnataka, 576104, India

**Keywords:** People living with HIV, PLWHA, HIV, AIDS, Biomarker, Saliva

## Abstract

**Background**: Biomarkers are measurable indicators of normal biological processes, which provide an objective assessment of the physiologic state of living systems. Saliva contains several biomarkers that serve as a diagnostic tool in health and disease. Evaluation of a multitude of salivary components could potentially predict the clinical outcome. This is especially critical in a chronic, potentially life-threatening condition like human immunodeficiency virus (HIV) infection. Scrupulous evaluation of relevant biomarkers could facilitate the early detection of HIV, determine the stage of infection and monitor the disease progression. Currently, there is a paucity of validated biomarkers in saliva predicting the disease progression in people living with HIV. In this scoping review, we aim to provide an overview of the available evidence on salivary markers associated with the progression of disease in people living with HIV.

**Methods: **The authors shall develop a tailored search strategy for each database using relevant keywords. We will search for eligible studies indexed in the following databases: MEDLINE, EMBASE, and the Cochrane Library, Cumulative Index to Nursing and Allied Health Literature (CINAHL) and gray literature. We will restrict the search to studies published in the English language. Following deduplication, all search results will be exported to the EPPI reviewer web, where two independent reviewers using a data extraction tool developed and pretested by the review authors will screen eligible studies. The result of this review will be reported using the Preferred Reporting Items for Systematic Review and Meta-Analyses Extension for Scoping Reviews (PRISMA-ScR) checklist and reporting guidelines.

**Discussion: **The proposed scoping review protocol will enable the identification and assessment of salivary biomarkers, which can predict disease progression in patients with HIV infection. The synthesis of evidence from this review will assist in improving our current understanding of biomarkers used to evaluate the progression of HIV infection.

## Introduction

The human immunodeficiency virus (HIV), belonging to the *Retroviridae* family, targets the body’s immune system ^1^. Since it has high affinity for the receptors present on the surface of CD4+ T-lymphocytes and macrophages, it makes a person vulnerable to infection ^2^. Infection of the target cell by HIV results in the production of progeny virions depleting the CD4+ lymphocytes and ensuing immunosuppression of the host. The pathophysiology of HIV involves a dynamic host-virus interaction, resulting in acquired immunodeficiency syndrome (AIDS) in severe cases ^3^. The incubation period for the virus is around 5–10 years in adults ^4, 5^. This broad interval between HIV infection and the development of symptoms can be attributed to several hosts and virus-related factors such as the development of new viral strains, the immune status of the host, as well as environmental cofactors ^6^. 

HIV viral load, CD4+ T-cell count in peripheral blood and quantitative measurements of soluble markers present in plasma, like neopterin, tumor necrosis factor-alpha (TNFα), interleukins (ILs), beta 2-microglobulin (B2M), soluble CD8, etc have been used as surrogate markers to assess the progression of HIV infection in patients. CD4+ T-cell count also evaluates the efficacy of the host’s immune response to antiretroviral therapy (ART) ^7– 10^. The onset of AIDS, which implies a progression of HIV infection, could be predicted accurately by monitoring the percentage of CD4+ T lymphocytes in the peripheral blood ^11^. However, in patients on ART, the CD4+ T-cell counts are not reliable markers to recognize virologic failure in the individual ^12^.

According to the National Institutes of Health (NIH), “a biomarker is an objectively measured and evaluated indicator of normal biologic processes, pathogenic processes, or pharmacologic responses to a therapeutic intervention” ^13^. Essentially, a biomarker can represent any entity and can exist as antibodies, microbes, DNA, RNA, lipids, metabolites, or proteins ^14^. These biomolecules provide crucial information that helps us understand the physiologic state of a biological system. Any alteration in their concentration, structure, function, or action within a biological system can be correlated with disease characteristics such as onset, progression, or even regression of the particular disease or a measure of the host response to foreign bodies ^15^. According to a review by Kanekar *et al.* in 2010, the clinical utility of biomarkers to assess the disease progression for HIV infection is inconclusive ^16^.

Saliva is a complex biological fluid that can mirror the body's health ^17^. It contains several biomolecules such as enzymes, hormones, antibodies, growth factors, antimicrobial constituents, etc. which can function as useful prognostic markers ^18^. A good salivary biomarker to detect the progression of HIV (with high sensitivity and specificity) would help clinicians and oral pathologists to monitor the deterioration of clinical condition in people living with HIV/AIDS (PLWHA) ^15, 19^.

The literature reveals the dearth of evidence on validated biomarkers ^16^. Much of the information on the topic is largely experimental, which has to be systematically compiled and objectively assessed. Therefore, this scoping review is aimed at synthesizing available evidence on salivary markers for disease progression in HIV infection.

## Objectives

- To identify pertinent salivary biomarkers consistent with the progression of HIV infection in HIV positive individuals
- To systematically review the existing literature on biomarkers in HIV to identify key concepts and gaps
- To assess the current levels of evidence, the quality of evidence and provide a synthesis of the currently available salivary biomarkers in HIV infection

## Methods

### Eligibility criteria

People diagnosed with HIV/AIDS (PLWHA) as per WHO clinical case definition is “an individual with HIV infection irrespective of the clinical stage (including severe or stage 4 clinical disease, also known as AIDS) confirmed by laboratory criteria according to country definitions and requirements”. We will include longitudinal studies that have measured outcomes of at least two different time points. Cross-sectional studies measuring clinical parameters at only one point will be excluded. Only studies that have reported an association between salivary biomarkers and change in the clinical measure will be included in our scoping review. Due to lack of sufficient resources, studies will be excluded if English language texts are not available.

We will not limit our inclusion based on age, gender, duration of HIV infection, ART status, or demography. Only studies that have reported measurable and quantifiable biological parameters associated with salivary biomarkers will be included. These parameters include, but are not limited to the presence of specific biomolecules, their biologic concentrations, specific gene-phenotype distribution in a population.

### Parameters by which a biomarker will be assessed

Its association with disease progression, its potential to be generalizable to PLWHA irrespective of their age, gender, sensitivity, specificity, reliability, ease of measurement, safety and acceptance to the patient. Additionally, it should reflect a true change in the clinical condition and remain unaffected by symptomatic treatment. We will exclude those studies that fail to meet the criterion of biomarker parameters mentioned. Studies that have not specified the type of surrogate marker used or include the objective measures of a particular biomarker or related only to specific opportunistic infections in HIV will be excluded.

### Protocol design

The methodological framework proposed by Arksey and O'Malley ^20^ and the methodological enhancement developed by Levac *et al.* ^21^ were referred to for this scoping review. The six-stage methodical framework for conducting a scoping review include: “(1) identifying the research question; (2) identifying relevant studies; (3) selecting studies; (4) charting the data; (5) collating, summarizing and reporting the results and (6) consulting with relevant stakeholders.”

### Stage 1: Identifying the research question

The research question developed by the research team in consultation with key stakeholders will address the role of salivary biomarkers in assessing the progression of disease in PLWHA. For this review, a quality indicator is ‘an explicitly and measurable item which act as building blocks in the assessment of care’.

### Stage 2: Identifying relevant studies

Search terms were finalized based on the feedback from the research team, subject experts, and extensive literature review. An experienced search scientist developed the search strategy and co-authors as per the Medline format and tailored to other databases and sources. The search strategy used in this scoping review included: (PLWHA OR PLHIV OR PLWH OR PLWA OR HIV OR (people living with HIV/AIDS) OR (people living with AIDS) OR (acquired AND (immunodeficiency OR immune‐deficiency OR immuno‐deficiency) AND syndrome) OR Immunocompromised OR immune-compromised OR Slim disease) AND ((HIV related oral lesions) OR (Periodontal disease) OR Periodontitis OR (periodontal infection) OR Xerostomia or (dry mouth) OR (salivary gland disease)) OR (Oral candidiasis) OR (hairy leukoplakia) OR (Kaposi sarcoma) OR (linear gingival erythema) OR (necrotizing ulcerative periodontitis) OR (aphthous ulcer) OR (wasting disease))) AND ((biological marker*) OR biomarker* OR saliva* OR biomolecule* OR (bacterial burden*) OR marker*)

The selected search terms will be searched in the title and/or abstract as well as subject headings keywords (eg, MeSH, EMTREE) as appropriate. We will include all articles from the beginning of the databases until October 2020. Only English language studies will be included. The search results from each database will be downloaded and imported onto Mendeley for the removal of duplicates. Following de-duplication, the remaining studies will be imported into the EPPI reviewer Web.

We will use the PICO (Population, Intervention, Control and Outcomes) strategy for formulating a foreground research question ( Table 1). Primary studies indexed in the following databases will be searched for inclusion in our review: MEDLINE, EMBASE, the Cochrane Library, Cumulative Index to Nursing and Allied Health Literature (CINAHL). The reference lists of all included studies will be hand searched for potentially relevant studies.

**Table 1.** PICO framework for the selection of studies.

Criteria	Inclusion criteria	Exclusion criteria
**Population**	People of all age groups and both genders diagnosed with HIV/ AIDS belonging to any region without any restriction on duration of the disease and staging	------
**Intervention/** **Exposure**	Any salivary biomarker, which is measurable and quantifiable	Studies reporting salivary biomarker, but not measured
**Comparison**	With or without any placebo or comparison of one biomarker with another	----
**Outcome**	Progression with respect to the staging of HIV or any other quantifiable outcome of the condition reported in the study including symptomatic improvements	------
**Study design**	Randomized control trials, non-randomized control trials, longitudinal studies with at least two time-point measurements, cohort studies, before and after comparison studies	Cross-sectional studies reporting the association between the salivary biomarker and clinical staging of HIV/AIDS at a single point in time
**Time frame**	Studies carried out till the search date irrespective of the duration of the study	-----

To ensure that all information pertinent to the research question is adequately captured, our search will include several grey literature sources from relevant databases (e.g. Grey Literature Report, OpenGrey, Web of Science Conference Proceedings). We will further conduct a targeted search of grey literature in the websites of organizations working on HIV/AIDS research on the local, provincial, national, and international levels. Any studies, reports, and conference abstracts identified through these databases, which are of relevance to this review, will be included.

### Stage 3: Study selection

We will undertake a two-step screening process to include all potentially relevant articles in this review: (1) title and abstract screening; (2) full-text screening. In the first stage of screening, two review authors (VD and PP) will independently screen the title and abstract of all retrieved citations for inclusion against a set of minimum inclusion criteria. These criteria will be determined by testing on a sample of abstracts before beginning the abstract review to ensure that they are robust enough to capture all studies pertinent to the primary objective. Articles will be included for full-text screening if either one or both of the review authors deem them relevant to the research question.

All the studies included in the T&A stage will be subject to full-text screening. In this step, both the investigators (VD and PP) will independently screen the full-text articles to assess if they meet the inclusion/exclusion criteria. We will calculate Cohen's κ statistics at both the T&A review stage and the full article review stage to determine inter-rater agreement. Studies will be reviewed another time if there is any discordance regarding the study eligibility. If there are further disagreements, it will be resolved through discussion with a third investigator (RR) until a consensus is reached. A flow diagram will be used to represent the inclusion and exclusion of retrieved studies

### Stage 4: Data collection

The research team will develop a data collection instrument to extract information from the included studies and to confirm study relevance. Study characteristics including publication year, publication type (eg, original research), study design, country, study setting, a specific biomarker used, statistical analysis performed, the association between biomarker tested and disease progression, the effect of therapeutic agents on biomarker changes, economic aspects and acceptability of biomarker, etc will be extracted (see Table 2). The research team will review and pretest the form to make sure that the data extraction form captures all the required information from the included studies accurately.

**Table 2.** Data charting form.

Parameters
Author and Year
Title of the study
Country
Aim/ objective of the study
Study design
Population
Settings
Sample size
Age
Duration of infection (length of infection)
Biomarker used
Biomarker classification
Method of biomarker obtained
Main findings of the study
Association between biomarker tested and disease progression
Does the biomarker associated with increase mortality
Correlation
Effect of therapeutic agents on biomarker changes
The method used for statistical analysis
Acceptability of biomarker
Conclusion
Confounders adjusted
Most relevant findings
Comment

Data from the included studies will be extracted independently by the two review authors (VD and PP) using the EPPI reviewer ^22^. To ensure a high degree of accuracy of the data extraction, we will compare the independently abstracted data of each reviewer. Both the review authors to ensure consistency in the extracted data will discuss any discrepancies identified in the collected data. The data will be compiled by the EPPI reviewer ^22^.

### Methodological quality

The quality tool developed by McGhee *et al.* in 2014 to assess the quality of surrogate biomarkers will be used to assess the overall methodological quality of the studies ^23^.

### Stage 5: Data summary and synthesis of results

We will synthesize the data narratively for each biomarker. All the outcomes stated in the studies will be reported. Additionally, we will present a summary of the range of outcomes where feasible.

We will assess the relationship between HIV infection and salivary biomarkers. We will report the effects of this relationship by variables reported in the studies, which were accounted for in the analysis. This review will further include a table of research implications, which will be extracted from each paper by research priorities. Additionally, we will report implications for clinical practice, where relevant. We will report the scoping review according to the PRISMA statement on reporting scoping reviews ^24^.

### Dissemination of information

The results of this scoping review will be disseminated through stakeholder meetings, conference presentations and peer-reviewed publications.

### Study status

The search strategy and final plan for data extraction are complete. Formal screening of search results against eligibility criteria is ongoing.

## Discussion

The proposed scoping review protocol will enable the identification and assessment of salivary biomarkers, which can predict disease progression in patients with HIV infection. Salivary components that mimic HIV infection progression can act as early predictors of the deteriorating clinical condition and serve as an alternative method to monitor the clinical condition. Moreover, its ease of extraction will correspond to greater compliance amongst patients when compared to other biofluids like blood. 

The synthesis of evidence from this review will assist in improving our current understanding of biomarkers used to evaluate HIV disease progression. This paper will be the pilot in a series of studies aimed at identifying and validating a salivary biomarker for the potential development of a point of care device, which can assess HIV infection progression.

## Data availability

### Underlying data

No data are associated with this article.
