# Supplementary material for: Salivary biomarkers associated with the progression of disease in people living with HIV: A scoping review protocol
Source: F1000Res. 2021 Sep 17;10:130. Originally published 2021 Feb 19. [Version 2] doi: 10.12688/f1000research.50813.2 (PMC10238815; doi:10.12688/f1000research.50813.2)
Supplement: Peer review history

# Peer review: Reviewer response for version 2

**DOI:** 10.5256/f1000research.77491.r173565

**Published:** 2023-06-02

**Authors:** Helen C. Steel

**Affiliations:** 1 Department of Immunology, Faculty of Health Sciences, University of Pretoria, Pretoria, South Africa

Prasad *et al.* propose identifying potential biomarkers present in saliva that can be associated with disease progression in PLWH. The Authors outline a scoping review protocol that will enable the identification and assessment of potential salivary biomarkers to be used to predict disease progression in these individuals.

 At present, biomarkers that are used to predict disease progression in PLWH are largely systemic in origin. The Authors propose that reliable saliva biomarkers could exclude the need for drawing blood from PLWH improving patient compliance, an important consideration with these individuals. In addition, this may, potentially, lead to the development of a point of care device to assess disease progression.

 Having said this, the Authors should be careful of dismissing the importance of VL, and CD4+ T-cell counts in assessing disease progression, and, importantly, treatment failure. These proposed biomarkers may not be an efficient, early marker of the latter. Biomarkers may also reflect other underlying conditions in these individuals. Please see Justice *et al.* (2018) ^1^ for clarity. ****Consider suggesting the use of these markers as an additional means of evaluating these aspects of the disease, possibly in the periods between VL and CD4+ T-cell counts.

 The rationale for, and objectives of, the study are clearly described and the study design is appropriate for the research question. Sufficient details of the methods are provided to allow replication by others.

 After reviewing the article I would like to give the status as "Approved" for this article as the comments or queries raised don't alter the quality of the review.

Is the study design appropriate for the research question?

Yes

Is the rationale for, and objectives of, the study clearly described?

Yes

Are sufficient details of the methods provided to allow replication by others?

Yes

Are the datasets clearly presented in a useable and accessible format?

Not applicable

Reviewer Expertise:

Immunology, infectious diseases, HIV, biomarkers. proteomics

I confirm that I have read this submission and believe that I have an appropriate level of expertise to confirm that it is of an acceptable scientific standard.

## References

[1] : Can Biomarkers Advance HIV Research and Care in the Antiretroviral Therapy Era?. *J Infect Dis* .2018;217(4) : 10.1093/infdis/jix586 521-528 10.1093/infdis/jix586 29165684PMC5853399

# Peer review: Reviewer response for version 1

**DOI:** 10.5256/f1000research.53901.r85305

**Published:** 2021-08-23

**Authors:** Purnima Madhivanan, Namoonga Mantina, Abidemi Okechukwu

**Affiliations:** 1 Department of Health Promotion Sciences, Mel and Enid Zuckerman College of Public Health, University of Arizona, Tucson, AZ, USA

This review was conducted by Namoonga Mantina and Abidemi Okechukwu under the guidance of Purnima Madhivanan. 

**General Review**

 This scoping review protocol was reviewed using the PRISMA-SCR checklist and the JBI Manual for Evidence Synthesis.

**General comments**

- In general, the methodology section of this protocol addresses most of the items in the PRISMA-SCR checklist.
- This protocol as it is written, however it communicates conflicting statuses to the reader­.  In some sections, it reads as a protocol and in other instances, it reads like the final report of the scoping review. In the methodology section, the authors noted that the search terms were finalized, however, within the same section, the authors noted that they “will use the PICO…strategy for formulating a foreground research question”. 

**Specific comments**

Title:

- Authors did not specify in the article title that the paper was a scoping review **protocol**. Leaves the reader to figure it out at the very end of the manuscript.

Introduction:

- While the salivary biomarkers for disease progression in HIV infection appears to be an under researched area, the area of study aligns well with the authors’ decision to conduct a scoping review. However, the introduction fails to explain why the questions and objectives of this study support a scoping review rather than a systematic review.
- Paragraph 2, first statement is missing a citation. Please include citations for the statement.
- Paragraph 5: The authors state that there is a “dearth of evidence of validated biomarkers”. As a general statement (encompassing all conditions), this seems inaccurate. If the authors mean to specifically refer to HIV/the condition being evaluated, then this should be added for clarification.
- The objectives of the study did not specify key components of the scoping review, which should be population or participants, concepts, and context. The authors might want to consider revising the objectives to specify each element. The PRISMA_SCR checklist can provide additional details.
-  In addition, Objective 2 is not specific to salivary biomarkers implying a much broader scope for review. This needs to be clarified along with clearly defining the limits of the scoping review. It seems this objective aims to explore all biomarkers related to HIV yet the stated premise of the review is to review salivary biomarkers. This objective seems beyond the scope of the review.
- The terminology to identify people living with HIV has be to used appropriately. We no longer use HIV positive individuals as a term to address people living with HIV.
- The case to evaluate saliva as a biomarker could be further explanation. From the information provided it seems that the only reason for choosing saliva is because CD4+ T-cell count aren’t reliable to detect virologic failure under ART. Has there been research to indicate that saliva would perform better under this condition and be worth investigating?

Methodology:

- Paragraph 1: Provide a reference for the clinical definition of PLWHA.
- Given the statement written by the authors that there is a dearth of studies on salivary biomarkers for monitoring HIV progression (paraphrased), limiting the search to studies published in English language may leave out important studies and limit the breadth of the scoping review. This is contrary to the very basis for conducting this review. Furthermore, the review, if limited to English language only, will bias the findings and the scope of the review will be further reduced.

- The third objective stated that “level of evidence” and “quality of evidence” will be assessed. How are the authors distinguishing these two? Furthermore, the methods section only details methodological quality. Furthermore, additional information on the tool being used would be helpful.

Is the study design appropriate for the research question?

Yes

Is the rationale for, and objectives of, the study clearly described?

Partly

Are sufficient details of the methods provided to allow replication by others?

Yes

Are the datasets clearly presented in a useable and accessible format?

Not applicable

Reviewer Expertise:

Systematic Reviews and Meta-analysis, Infectious Diseases, HIV/AIDS, Diagnostics for developing world

We confirm that we have read this submission and believe that we have an appropriate level of expertise to confirm that it is of an acceptable scientific standard, however we have significant reservations, as outlined above.

# Response

**Published:** 2021-09-17

**Authors:** Raghu Radhakrishnan

**Affiliations:** Manipal Academy of Higher Education, India

**Dear Reviewers,**

**The authors thank the reviewer for their kind comments. We are pleased to respond to the reviewer's comments.**

Title:

- Authors did not specify in the article title that the paper was a scoping review protocol. Leaves the reader to figure it out at the very end of the manuscript. - **Changes have been made in the manuscript and re uploaded**

Introduction:

- While the salivary biomarkers for disease progression in HIV infection appears to be an under researched area, the area of study aligns well with the authors’ decision to conduct a scoping review. However, the introduction fails to explain why the questions and objectives of this study support a scoping review rather than a systematic review. **– A sentence in this regard is now added at the end of Introduction.**
- Paragraph 2, first statement is missing a citation. Please include citations for the statement. **- Changes have been made in the manuscript and re uploaded.**

- Paragraph 5: The authors state that there is a “dearth of evidence of validated biomarkers”. As a general statement (encompassing all conditions), this seems inaccurate. If the authors mean to specifically refer to HIV/the condition being evaluated, then this should be added for clarification. **- Changes have been made in the manuscript and re uploaded.**

- The objectives of the study did not specify key components of the scoping review, which should be population or participants, concepts, and context. The authors might want to consider revising the objectives to specify each element. The PRISMA_SCR checklist can provide additional details.

**Objectives:**

1. **To identify pertinent salivary biomarkers consistent with the progression of HIV infection in HIV positive individuals.**
2. **To systematically review the existing literature on biomarkers in HIV to identify key concepts and gaps.**
3. **To assess the current levels of evidence the quality of evidence and provide a synthesis of the currently available salivary biomarkers in HIV infection.**

- **P - HIV positive individuals**
- **E - Salivary biomarker**
- **O - progression of the disease**
- In addition, Objective 2 is not specific to salivary biomarkers implying a much broader scope for review. This needs to be clarified along with clearly defining the limits of the scoping review. It seems this objective aims to explore all biomarkers related to HIV yet the stated premise of the review is to review salivary biomarkers. This objective seems beyond the scope of the review. **- Changes have been made in the manuscript and re uploaded.**

- The terminology to identify people living with HIV has be to used appropriately. We no longer use HIV positive individuals as a term to address people living with HIV **– Changes have been made in the manuscript and re uploaded.**

- The case to evaluate saliva as a biomarker could be further explanation. From the information provided it seems that the only reason for choosing saliva is because CD4+ T-cell count aren’t reliable to detect virologic failure under ART. Has there been research to indicate that saliva would perform better under this condition and be worth investigating? **-** **We have added more explanation in the introduction section in this regard.**

Methodology:

- Paragraph 1: Provide a reference for the clinical definition of PLWHA. **- Changes have been made in the manuscript and re uploaded.**

- Given the statement written by the authors that there is a dearth of studies on salivary biomarkers for monitoring HIV progression (paraphrased), limiting the search to studies published in English language may leave out important studies and limit the breadth of the scoping review. This is contrary to the very basis for conducting this review. Furthermore, the review, if limited to English language only, will bias the findings and the scope of the review will be further reduced. **- This is beyond the scope of the current review**

- The third objective stated that “level of evidence” and “quality of evidence” will be assessed. How are the authors distinguishing these two? Furthermore, the methods section only details methodological quality. Furthermore, additional information on the tool being used would be helpful. **– Changes have been made in the manuscript and re uploaded.**

# Peer review: Reviewer response for version 1

**DOI:** 10.5256/f1000research.53901.r87039

**Published:** 2021-06-21

**Authors:** Pascale Ondoa

**Affiliations:** 1 African Society for Laboratory Medicine, Addis Ababa, Ethiopia

***30/06/2021 - Updated review (** **In response to the author’s comments):***

*I acknowledged that I mistakenly reviewed the paper as a Systematic Review Study Protocol instead of a Scoping Review Study Protocol.*

*I still think that the paper has many gaps and should not be accepted as is. The authors should revise their piece and include the requested information. Looking at the JBI manual for scoping review https://wiki.jbi.global/display/MANUAL/11.2.7+Data+extraction, https://guides.library.unisa.edu.au/ScopingReviews/Protocol  the minimum information one expects to see in a protocol are not provided.*

**21/06/2021 - Original review:**

 This paper intends to introduce a systematic review of available data on markers for HIV disease progression in saliva.

 The introduction is brief and does not correctly bring the rationale for identifying markers of disease progression. Viral Load is considered a reliable and early indicator of virological failure in patients receiving ART and is recommended by the WHO. Alternative markers might be useful, but we need to understand why. The discussion on the lesser value of CD4 to swiftly identify disease progression is an old one. Maybe the authors would like to develop assays that do not require blood collection and molecular testing? Then it should be more clearly outlined.

 In addition to the poor background information, a systematic review protocol is supposed to provide a detailed plan of the methodology and analysis beforehand. Should the research question not be defined at this stage? The following are missing in the paper:

- The search terms (key words) for the search.
- The time frame of coverage.
- The search strategy.
- The extraction of data.
- The outcome measures: how is a pertinent salivary marker defined?
- The strategy to handle biases

Is the study design appropriate for the research question?

No

Is the rationale for, and objectives of, the study clearly described?

No

Are sufficient details of the methods provided to allow replication by others?

No

Are the datasets clearly presented in a useable and accessible format?

No

Reviewer Expertise:

Laboratory science, HIV immunology and virology, Laboratory system strengthening, diagnostics

I confirm that I have read this submission and believe that I have an appropriate level of expertise to state that I do not consider it to be of an acceptable scientific standard, for reasons outlined above.

# Response

**Published:** 2021-06-29

**Authors:** Raghu Radhakrishnan

**Affiliations:** Manipal Academy of Higher Education, India

Dear Reviewer

 Thank you for your comments. As a corresponding author, I am pleased to respond to your comments. Please find my responses as follows.

**Query - 1: **This paper intends to introduce a systematic review of available data on markers for HIV disease progression in saliva. This paper intends to introduce a systematic review of available data on markers for HIV disease progression in saliva

**Response - 1:**This paper is a scoping review and not a systematic review. This paper aims to ‘map the literature of available data on markers for HIV disease progression in saliva and to provide an opportunity to identify key concepts; gaps in the research; and types and sources of evidence to inform practice, policymaking, and research’ (Daudt *et al.*) 

**Query - 2: **The introduction is brief and does not correctly bring the rationale for identifying markers of disease progression. Viral Load is considered a reliable and early indicator of virological failure in patients receiving ART and is recommended by the WHO. Alternative markers might be useful, but we need to understand why. The discussion on the lesser value of CD4 to swiftly identify disease progression is an old one. Maybe the authors would like to develop assays that do not require blood collection and molecular testing? Then it should be more clearly outlined.

**Response - 2:** We agree with the reviewer that “Viral Load is considered a reliable and early indicator of virological failure in patients receiving ART and is recommended by the WHO”. However, they might not be readily available in resource-limited settings (Ford *et al*; Ferreyra *et al.*)

**Query - 3:** In addition to the poor background information, a systematic review protocol is supposed to provide a detailed methodology and analysis plan beforehand. Should the research question not be defined at this stage? The following are missing in the paper:

**Response - 3: **This is a protocol for a scoping review and not a systematic review and the following information are quoted from the manuscript. The search terms (keywords) for the search, The time frame of coverage. I wish to inform the reviewer that this paper intends to introduce a systematic review of available data on markers for HIV disease progression in saliva.

 References:

 Daudt HM, van Mossel C, Scott SJ. Enhancing the scoping study methodology: a large, inter-professional team's experience with Arksey and O'Malley's framework. BMC Medical Research Methodology. 2013;13:48. DOI: 10.1186/1471-2288-13-48.

 Ford N, Meintjes G, Pozniak A, Bygrave H, Hill A, Peter T, *et al.* The future role of CD4 cell count for monitoring antiretroviral therapy. Lancet Infect Dis. Elsevier Ltd; 2015;15(2):241–7.

 Ferreyra C, Yun O, Eisenberg N, Alonso E, Khamadi AS, Mwau M, *et al.* Evaluation of Clinical and Immunological Markers for Predicting Virological Failure in an HIV/AIDS Treatment Cohort in Busia, Kenya. PLoS One. 2012;7(11)

# Response

**Published:** 2021-07-01

**Authors:** Raghu Radhakrishnan

**Affiliations:** Manipal Academy of Higher Education, India

Dear Reviewer 

 We acknowledge the reviewer’s comments and thank F1000 for inviting Dr. Pascale Ondoa to review our manuscript. The authors would like to humbly respond to the information that the reviewer has sought. For ease of understanding, we would like to draw information from our manuscript for each JBI criteria and presented it below for your kind consideration. 

**(1) An introduction detailing:**

- **Definitions**
  - Per our manuscript: “According to the National Institutes of Health (NIH), “a biomarker is an objectively measured and evaluated indicator of normal biologic processes, pathogenic processes, or pharmacologic responses to a therapeutic intervention”13. Essentially, a biomarker can represent any entity and can exist as antibodies, microbes, DNA, RNA, lipids, metabolites, or proteins”
- **Overall review objective**
  - From the manuscript:
  - “Objectives -
    - To identify pertinent salivary biomarkers consistent with the progression of HIV infection in HIV positive individuals
    - To systematically review the existing literature on biomarkers in HIV to identify key concepts and gaps
    - To assess the current levels of evidence, the quality of evidence and provide a synthesis of the currently available salivary biomarkers in HIV infection”
- **Aim:**
  - Per our manuscript: “aimed at synthesizing available evidence on salivary markers for disease progression in HIV infection”.
- **Details of any preliminary searches undertaken**
- **Explanation of the need for review**
  - Per our manuscript: "The literature reveals the dearth of evidence on validated biomarkers16. Much of the information on the topic is largely experimental, which has to be systematically compiled and objectively assessed."
- **Eligibility criteria (with contextualization and rationalisation)**
  - From the manuscript: “People diagnosed with HIV/AIDS (PLWHA) as per WHO clinical case definition is “an individual with HIV infection irrespective of the clinical stage (including severe or stage 4 clinical disease, also known as AIDS) confirmed by laboratory criteria according to country definitions and requirements”.
  - We will include longitudinal studies that have measured outcomes of at least two different time points. Cross-sectional studies measuring clinical parameters at only one point will be excluded. Only studies that have reported an association between salivary biomarkers and change in the clinical measure will be included in our scoping review. Due to a lack of sufficient resources, studies will be excluded if English language texts are not available.
  - We will not limit our inclusion based on age, gender, duration of HIV infection, ART status, or demography. Only studies that have reported measurable and quantifiable biological parameters associated with salivary biomarkers will be included. These parameters include, but are not limited to the presence of specific biomolecules, their biologic concentrations, specific gene-phenotype distribution in a population.

**(2) Sample Search Strategy**

- From the manuscript: “The search strategy used in this scoping review included: (PLWHA OR PLHIV OR PLWH OR PLWA OR HIV OR (people living with HIV/AIDS) OR (people living with AIDS) OR (acquired AND (immunodeficiency OR immune‐deficiency OR immuno‐deficiency) AND syndrome) OR Immunocompromised OR immune-compromised OR Slim disease) AND ((HIV related oral lesions) OR (Periodontal disease) OR Periodontitis OR (periodontal infection) OR Xerostomia or (dry mouth) OR (salivary gland disease)) OR (Oral candidiasis) OR (hairy leukoplakia) OR (Kaposi sarcoma) OR (linear gingival erythema) OR (necrotizing ulcerative periodontitis) OR (aphthous ulcer) OR (wasting disease))) AND ((biological marker*) OR biomarker* OR saliva* OR biomolecule* OR (bacterial burden*) OR marker*)”

**3) Explanation of search approach, including:**

- **Which black and grey literature will be searched?**
  - Per manuscript: “To ensure that all information pertinent to the research question is adequately captured, our search will include several grey literature sources from relevant databases (e.g. Grey Literature Report, OpenGrey, Web of Science Conference Proceedings). We will further conduct a targeted search of grey literature on the websites of organizations working on HIV/AIDS research on the local, provincial, national, and international levels. Any studies, reports, and conference abstracts identified through these databases, which are of relevance to this review, will be included.”
- **Justification for choices**
  - From the manuscript: “To ensure that all information pertinent to the research question is adequately captured”

**(4) Study selection process, including resolving disagreements between reviewers**

- From the manuscript: “We will undertake a two-step screening process to include all potentially relevant articles in this review: (1) title and abstract screening; (2) full-text screening.
- In the first stage of screening, two review authors (VD and PP) will independently screen the title and abstract of all retrieved citations for inclusion against a set of minimum inclusion criteria. These criteria will be determined by testing on a sample of abstracts before beginning the abstract review to ensure that they are robust enough to capture all studies pertinent to the primary objective. Articles will be included for full-text screening if either one or both of the review authors deem them relevant to the research question.
- All the studies included in the T&A stage will be subject to full-text screening. In this step, both the investigators (VD and PP) will independently screen the full-text articles to assess if they meet the inclusion/exclusion criteria. We will calculate Cohen's κ statistics at both the T&A review stage and the full article review stage to determine inter-rater agreement. Studies will be reviewed another time if there is any discordance regarding the study eligibility. If there are further disagreements, they will be resolved through discussion with a third investigator (RR) until a consensus is reached. A flow diagram will be used to represent the inclusion and exclusion of retrieved studies”

**(5) A draft charting table/form for data extraction and accompanying explanation**

- Data charting form: Table number 2.
  - From the manuscript: “The research team will develop a data collection instrument to extract information from the included studies and to confirm study relevance. Study characteristics including publication year, publication type (eg, original research), study design, country, study setting, a specific biomarker used, statistical analysis performed, the association between biomarker tested and disease progression, the effect of therapeutic agents on biomarker changes, economic aspects and acceptability of biomarker, etc will be extracted (see Table 2). The research team will review and pretest the form to make sure that the data extraction form captures all the required information from the included studies accurately.”

**(6) How results and data will be presented (e.g. draft chart, figure or table)**

- From the manuscript:
  - “We will synthesize the data narratively for each biomarker. All the outcomes stated in the studies will be reported. Additionally, we will present a summary of the range of outcomes where feasible.
  - We will assess the relationship between HIV infection and salivary biomarkers.
  - We will report the effects of this relationship by variables reported in the studies, which were accounted for in the analysis.
  - This review will further include a table of research implications, which will be extracted from each paper by research priorities.
  - Additionally, we will report implications for clinical practice, where relevant.
  - We will report the scoping review according to the PRISMA statement on reporting scoping reviews24.”

**(7) Data Extraction:**

- We have grouped the information and presented it across the JBI charting form for your reference and ease in table 2
  - Author(s): From the manuscript: “Author”.
  - Year of publication: From the manuscript: “Year of publication”.
  - Origin/country of origin (where the source was published or conducted): From the manuscript: “country”.
  - Aims/purpose: From the manuscript: “Aim/ objective of the study”.
  - Population and sample size within the source of evidence (if applicable): From the manuscript: “population, sample size”.
  - Methodology/methods: From the manuscript: “Study design, settings, age, duration of infection”.
  - Intervention type, comparator, and details of these (e.g. duration of the intervention) (if applicable). Duration of the intervention (if applicable): From the manuscript: “biomarker used, biomarker classification.
  - Outcomes and details of these (e.g. how measured) (if applicable) (method of biomarker obtained): From the manuscript: “Main findings of the study, the association between biomarker tested and disease progression, does the biomarker associated with increase mortality, Correlation, Effect of therapeutic agents on biomarker changes, The method used for statistical analysis, Acceptability of biomarker.
  - Key findings that relate to the scoping review question/s.: From the manuscript: “Conclusion, Confounders adjusted, most relevant findings”.

The authors once again wish to place on record our sincere gratitude and appreciation to the editorial board for providing us an opportunity to contribute to F1000.

 Thanking you.

 Kind regards

 Raghu

# Peer review: Reviewer response for version 1

**DOI:** 10.5256/f1000research.53901.r85113

**Published:** 2021-05-18

**Authors:** Sudhir Prabhu

**Affiliations:** 1 Department of Community Medicine, Father Muller Medical College, Mangalore, India

The review article planned is clinically relevant and scientifically sound without any major errors.

 However the following minor technical issues might need to be addressed for the readers mainly for better clarity and understanding the purpose of the review:

 1. Objective 2 mentions "to identify key concepts and gaps" it is not clear whether it is with respect to existing biomarkers or only salivary biomarkers or both. Are the authors trying to convey through this review that due to limitations of biomarkers (lab/clinical), salivary biomarkers could fill that knowledge gap in the natural history of HIV?

 2. Outcome variables in HIV is normally assessed using Clinical, Immunological (CD4) and Virological (viral load) progression, when the authors mention outcomes in the PICO framework to be "quantifiable" does it mean only CD4 and viral load, both of them couldn't be seen in MeSH terms.

 3. Also progression of HIV which is mentioned as clinical staging, can be better mentioned as WHO staging and could be cited. Were there any other staging used for monitoring?

 After reviewing the article I would like to give the status as **"Approved"** for this article as the comments or queries raised don't alter the quality of the review. Even if the authors don't comply with the changes suggested here, the scientific and ethical validity of the systematic review planned by them will not change.

Is the study design appropriate for the research question?

Yes

Is the rationale for, and objectives of, the study clearly described?

Yes

Are sufficient details of the methods provided to allow replication by others?

Yes

Are the datasets clearly presented in a useable and accessible format?

Yes

Reviewer Expertise:

Community medicine, vaccines, non-communicable diseases, maternal and child health, HIV, infectious diseases

I confirm that I have read this submission and believe that I have an appropriate level of expertise to confirm that it is of an acceptable scientific standard.

# Response

**Published:** 2021-05-23

**Authors:** Raghu Radhakrishnan

**Affiliations:** Manipal Academy of Higher Education, India

Dear Reviewer

 The authors thank the reviewer for their kind comments. We are pleased to respond to the reviewer's comments.

**Comment:** Objective 2 mentions "to identify key concepts and gaps" it is not clear whether it is for existing biomarkers or only salivary biomarkers or both. Are the authors trying to convey through this review that due to limitations of biomarkers (lab/clinical), salivary biomarkers could fill that knowledge gap in the natural history of HIV?.

**Response**: We are systematically reviewing the existing literature only on salivary biomarkers in HIV.

**Comment:** Outcome variables in HIV are normally assessed using Clinical, Immunological (CD4) and Virological (viral load) progression, when the authors mention outcomes in the PICO framework to be "quantifiable" does it mean only CD4 and viral load, both of them couldn't be seen in MeSH terms.

**Response: **Often scientists do not include outcome measures in the search strategy, because many abstracts do not contain a description of these outcome measures. (Laboratory Animals 2012;46: 24–31. DOI: 10.1258/la.2011.01108)

**Comment: **The progression of HIV, which is mentioned as clinical staging, can be better mentioned as WHO staging and cited. Was there any other staging used for monitoring?

**Response:** Progression was with the immunological and clinical staging of HIV ( https://www.who.int/hiv/pub/guidelines/HIVstaging150307.pdf).
